# Computing Generalized Matrix Inverse on Spiking Neural Substrate

**DOI:** 10.3389/fnins.2018.00115

**Published:** 2018-03-13

**Authors:** Rohit Shukla, Soroosh Khoram, Erik Jorgensen, Jing Li, Mikko Lipasti, Stephen Wright

**Affiliations:** ^1^Department of Electrical and Computer Engineering, University of Wisconsin-Madison, Madison, WI, United States; ^2^Department of Electrical and Computer Engineering, Georgia Institute of Technology, Atlanta, GA, United States; ^3^Department of Computer Sciences, University of Wisconsin-Madison, Madison, WI, United States

**Keywords:** spiking neural networks, TrueNorth, matrix inversion, Hopfield neural network, neuromorphic computing, stochastic computing

## Abstract

Emerging neural hardware substrates, such as IBM's TrueNorth Neurosynaptic System, can provide an appealing platform for deploying numerical algorithms. For example, a recurrent Hopfield neural network can be used to find the Moore-Penrose generalized inverse of a matrix, thus enabling a broad class of linear optimizations to be solved efficiently, at low energy cost. However, deploying numerical algorithms on hardware platforms that severely limit the range and precision of representation for numeric quantities can be quite challenging. This paper discusses these challenges and proposes a rigorous mathematical framework for reasoning about range and precision on such substrates. The paper derives techniques for normalizing inputs and properly quantizing synaptic weights originating from arbitrary systems of linear equations, so that solvers for those systems can be implemented in a provably correct manner on hardware-constrained neural substrates. The analytical model is empirically validated on the IBM TrueNorth platform, and results show that the guarantees provided by the framework for range and precision hold under experimental conditions. Experiments with optical flow demonstrate the energy benefits of deploying a reduced-precision and energy-efficient generalized matrix inverse engine on the IBM TrueNorth platform, reflecting 10× to 100× improvement over FPGA and ARM core baselines.

## 1. Introduction

Recent advances in neuromorphic engineering (Schuman et al., 2017) have motivated the development of neural hardware substrates that are tailored to loosely emulate computations that happen in a human brain with extremely low power and efficiency. Examples include IBM TrueNorth Neurosynaptic System (Merolla et al., 2014), NeuroFlow (Cheung et al., 2016), Neurogrid (Benjamin et al., 2014), SpiNNaker (Furber et al., 2014), and the BrainScaleS project (Schemmel et al., 2008), all of which are implemented using Si CMOS. While Si CMOS is the prevailing technology, the slowdown in transistor scaling has led to broad interest in spiking neural network substrates that exploit the unique properties of emerging nonvolatile memory such as Narayanan et al. (2017) and RRAM (Goux et al., 2013). Due to the close match between the algorithmic requirements and the underlying hardware architecture, such designs have the potential to achieve much better computational efficiency than the conventional Si-CMOS based designs.

In spite of the radically differing hardware implementations of these neural network substrates, many of them share an inherent design principle: converting input signal amplitude information into a rate-coded spike train and performing parallel operations of dot-product computations on these spike trains, based on synaptic weights stored in the memory array. These similarities also result in a set of common challenges during practical implementation, especially when using them as computing substrates for applications with a mathematical algorithmic basis. These challenges include a restricted range of input values and the limited precision of synaptic weights and inputs. Since a value is encoded in unary spikes over time (i.e., as a firing rate), each individual input and variable must take a value in the range [0, 1]. Furthermore, the precision of the encoded value is directly proportional to the size of the evaluation window, which, for reasons of efficiency, is typically limited to a few hundred spikes. Finally, because of hardware cost, synaptic weights can be implemented only by a limited number of memory bits, resulting in limited precision. For instance, IBM's TrueNorth supports 9-bit weight values, where most significant indicates sign of the weights.

Mapping existing algorithms to these substrates requires the designer to choose a strategy for quantizing inputs and weights carefully, so that the range limitations are not violated (i.e., values represented by firing rates do not saturate), while maintaining sufficient precision. Prior work notes these challenges, but typically presents only *ad hoc* solutions that choose scaling factors and quantization strategies based on empirical measurements that can guarantee correct operation for the tested scenarios, but provides no guarantees in the general case (Jin et al., 2008; Shukla et al., 2017). Error analysis for feedforward networks appear in Hopkins and Furber (2015), but omits recurrent networks and range analysis.

In contrast, this paper develops a rigorous mathematical model that enables a designer to map numerical algorithms to these substrates and to reason quantitatively about the range and precision of the computation taking place in the neural substrate. Our mathematical framework can be applied to a wide range of problems in linear optimization running on neural substrates with diverse constraints. The model is validated empirically by constructing input matrices with random values and computing matrix inverse using a recurrent Hopfield neural-network-based linear solver. Our results show that the scaling factor and error bounds derived by the mathematical model hold for this application under a broad range of input conditions. We report the computing resources and power numbers for real-time applications, and quantify how the errors and inefficiencies can be addressed to enable practical deployment of the Hopfield linear solver.

Prior work by authors in Shukla et al. (2017) showed how these linear solvers can be used in a variety of robotic applications, such as computing transformation matrices. They have discussed how weights can be encoded on TrueNorth, as in section 2.3.1 of this paper, and have reported experiments using a weight-encoded scheme. However, Shukla et al. (2017) does not show any mathematical model of the Hopfield linear solver (section 2.2 of this paper) and does not contain results about error bounds (section 2.5). There was no discussion about Hopfield weights being encoded as spiking inputs (section 2.3.2) and no description regarding algorithmic steps required to implement the linear solver on a spiking neural substrate like TrueNorth (section 2.4). All these issues are addressed in this paper. Additionally, section 3 of this paper presents a more thorough analysis of experiments with respect to dynamic inputs, where the Hopfield network weights are represented as spiking inputs.

## 2. Materials and methods

### 2.1. Background

This section describes how a system of linear equations can be solved using a recurrent Hopfield neural network, and shows how such a solver can be used in applications such as target tracking and optical flow. These example applications have been successfully deployed on TrueNorth (Shukla et al., 2017), by applying the analytical framework presented in this paper to probe its range and precision requirements.

#### 2.1.1. Solving linear systems with a hopfield network

A linear equations solver is used to solve matrix equations of the form *AX* = *B*. More generally, to accommodate the case of infeasible systems, we obtain *X* from the following linear least squares problem:

(1) $$\begin{array}{l} \underset{X}{\min}\frac{1}{2}\|AX-B{\|}_{F}^{2}, \end{array}$$

where *A* is an *M* × *N* matrix with *M* ≥ *N*, *B* is a matrix of dimension *M* × *P*, and ‖·‖_*F*_ is the Frobenius norm. Note that the problem decomposes by columns of *B*, so we can write Equation (1) equivalently as

(2) $$\begin{array}{l} \underset{{\left[X\right]}_{\cdot j}}{\min}\frac{1}{2}\|A{\left[X\right]}_{\cdot j}-B_{\cdot j}{\|}_{2}^{2},\;j=1,2,\ldots ,P, \end{array}$$

where [*X*]_·*j*_ denotes the *j*th column of the matrix *X*. By setting the the gradient of Equation (1) to zero, we obtain the following “normal equations:”

(3) $$\begin{array}{l} A^{T}AX=A^{T}B. \end{array}$$

This system can be solved by the following stationary iterative process:

(4) $$\begin{array}{l} X_{k+1}=X_{k}+\alpha \left(-A^{T}AX_{k}+A^{T}B\right) \end{array}$$

(5) $$\begin{array}{l} =\left(I-\alpha A^{T}A\right)X_{k}+\alpha A^{T}B, \end{array}$$

(6) $$\begin{array}{l} \text{where }X_{0}:=\alpha A^{T}B, \end{array}$$

and α is a positive steplength. (This process can also be thought of as a steepest descent method applied to the optimization problem Equation (2). For convergence of this process, we require

(7) $$\begin{array}{l} 0<\alpha <\frac{2}{\lambda_{\max}\left(A^{T}A\right)}, \end{array}$$

where $\lambda_{\max}\left(A^{T}A\right)$ denotes the maximum eigenvalue of *A*^*T*^*A*. This condition ensures that all eigenvalues of (*I* − α*A*^*T*^*A*) lie in the interval (−1, 1]. (If *A* is rank deficient, *A*^*T*^*A* is singular, so some eigenvalues of (*I* − α*A*^*T*^*A*) will be 1 in this case.) See Ben-Israel and Charnes (1963) for a proof of convergence.

We can map this process Equation (4) to a recurrent Hopfield network cleanly by rewriting it as follows:

(8) $$\begin{array}{l} X_{k+1}=W_{\text{hop}}X_{k}+W_{\text{ff}}B,k=0,1,2,\ldots , \end{array}$$

where

(9a) $$\begin{array}{l} W_{\text{hop}}:=I-\alpha A^{T}A, \end{array}$$

(9b) $$\begin{array}{l} W_{\text{ff}}:=\alpha A^{T}. \end{array}$$

**Figure 1:**
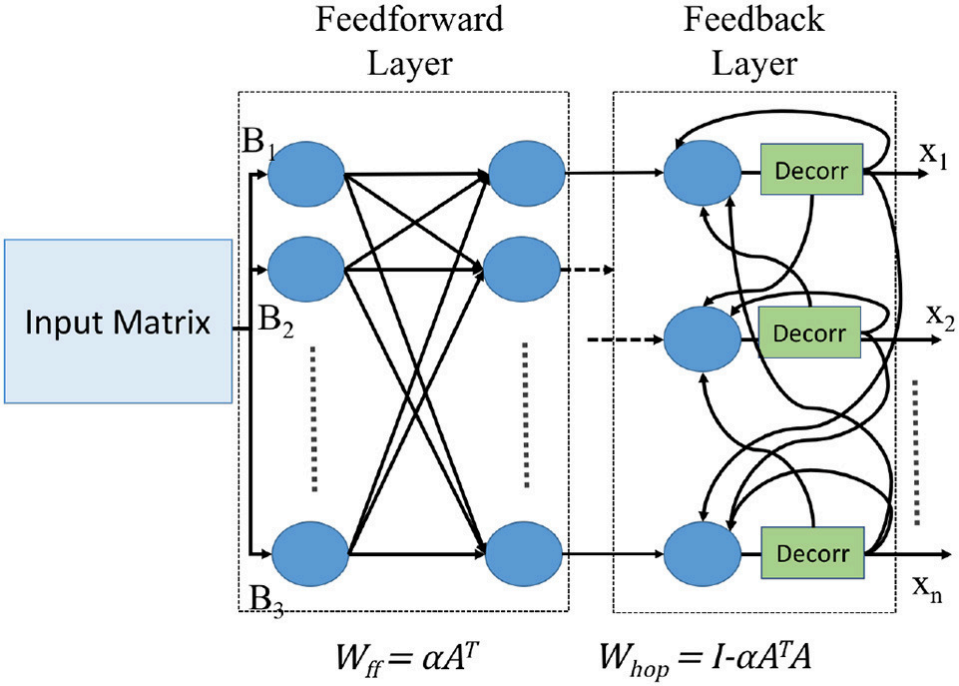
Neural network architecture of Hopfield linear solver.

The Hopfield neural network architecture for implementing Equation (8) is shown in Figure 1.

This elementary derivation shows that we can solve arbitrary systems of linear equations (which we refer to also as “matrix division”) directly in a recurrent neural network by loading synaptic weight coefficients *W*_ff_ and *W*_hop_ derived from *A* and α into the neural network, and connecting the inputs *b* and recurrent outputs *X*_*k*+1_ appropriately. The weight matrix *W*_ff_ serves as the feedforward weight for the input matrix *B*, while *W*_hop_ serves as the weight for the recurrent part for the values *X*_*k*_.

We have implemented prototypes for two different classes of applications. The first prototype is for applications in which Hopfield network weights are hard-coded on TrueNorth, while the second prototype is for applications in which Hopfield network weights are encoded as dynamic spike trains.

#### 2.1.2. Hopfield network weights hard-coded on TrueNorth

**Figure 2:**
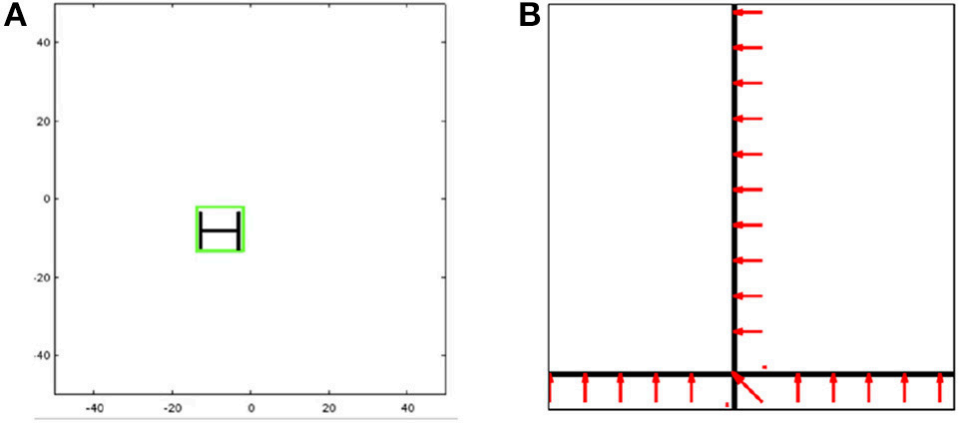
Illustration of **(A)** target tracking and **(B)** optical flow.

For the first class of applications, we consider a typical target tracking scenario, shown in Figure 2A. We are repeating the same steps that was published in Shukla et al. (2017) to replicate the target tracking experiment. Here, the features do not change often, remaining fixed for long time periods, during which the matrix *A* in Equation (3) stays the same. The Hopfield network weights *W*_ff_ and *W*_hop_ can be precomputed and hard-coded onto TrueNorth board for use.

In target tracking, a real-time video input is preprocessed to extract features (e.g., edges of particular orientations) to form a feature set. This feature set is then compared against a set of templates to identify objects of interest, with the goal of tracking the objects in the image frame as they move in three dimensions. As a proof of concept, we have chosen a very simple image whose feature set consists of just three edges similar in appearance to the letter H. To determine size and placement of the bounding box for the tracked image, we utilize the theory of affine transforms which shows that a current image *B* can be matched to its template *A* via an affine transformation *X* using a matrix multiplication *AX* = *B*, as long as the image has been transformed only with respect to the template in scale, rotation, or 2D translation. (A similar self-learning visual architecture was investigated in Shukla and Lipasti, 2015). By employing matrix division implemented in the recurrent Hopfield network (Equation 8), we can derive the affine transform *X* that maps the current image input *b* to the template *A*, thus determining the scale and the horizontal and vertical transformations from the matrix *X*.

#### 2.1.3. Hopfield network weights as dynamic spiking input

We investigated optical flow as our second application, shown in Figure 2B. We are repeating the same steps that was published in Shukla et al. (2017) to replicate the optical flow experiment. In optical flow, the matrix *A* might change at certain (frequent and regular) time intervals, so it is not viable to hard-code weight assignment on TrueNorth. To implement a more dynamic pseudoinverse calculator, we make use of concepts from stochastic computing (Gaines, 1967; Alaghi and Hayes, 2013) and demonstrate the versatility of spiking neural substrates.

Our demonstration is similar to the one reported in Esser et al. (2013) where the horizontal bar is continuously moving upwards and the vertical bars are moving to the left of the screen. In this prototype, we demonstrate that the direction of movement of the bars can be reported without any error, and the speed at which the two bars move apart can be determined approximately. This is done by solving for *X* in the equation *AX* = *B*, where the matrix *A* contains partial derivatives of initial image frame with respect to *x* and *y* directions, and the vector *B* contains partial derivatives of pixel positions between the initial image frame and the image frame at time *t*. The solution matrix *X* indicates the speed and direction of the image pixels.

#### 2.1.4. Computing in spikes

IBM TrueNorth is a biologically-inspired architecture that performs computations using spiking neurons. Input values are represented in a stochastic time-based coding, in which the probability of occurrence of a spike at a particular time tick is directly proportional to the input value. Since the computation values are represented as spike trains, designers are faced with two key issues in mapping algorithms to these spiking neural substrate.

1. Signed computations on spiking neural network substrates that have input values represented as rate based encoding must be performed by splitting all numbers (and intermediate results) into positive and negative parts.
2. Data representation is limited by maximum frequency of spikes. To represent different values within a matrix, we need to scale all quantities so that no number exceeds this maximum frequency.

**Figure 3:**
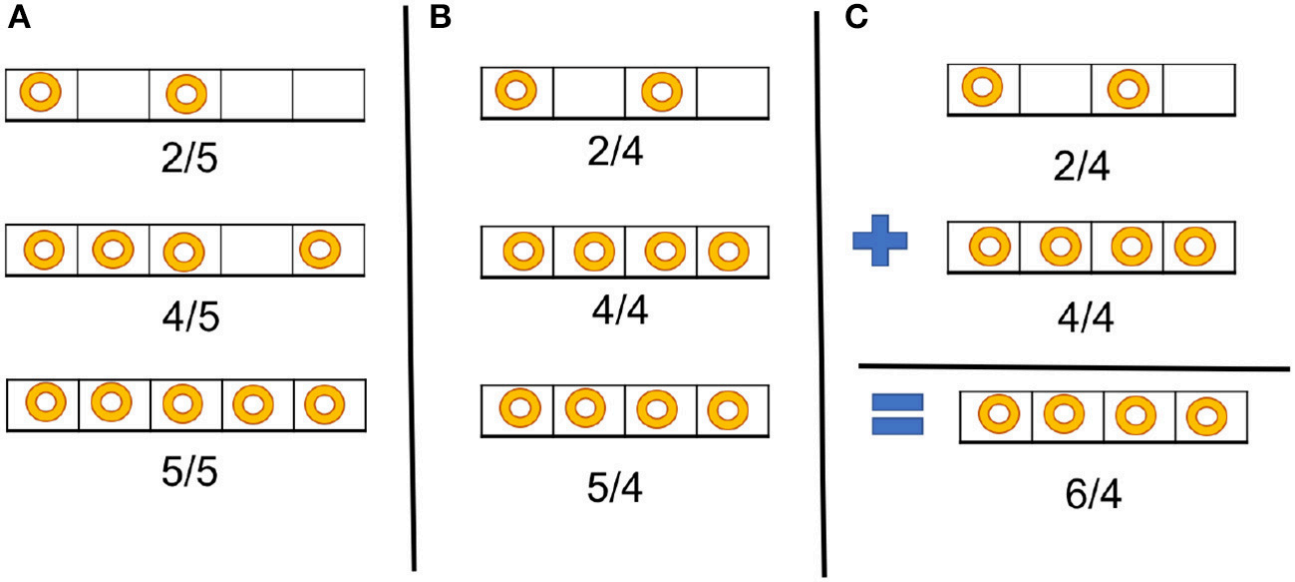
Example illustrating the importance of proper scaling for spike-based computation. **(A)** All three values of a vector are scaled properly. **(B)** Inappropriate scaling: two values (4 and 5) are represented by the same spike rate. **(C)** Addition of two numbers that are scaled properly, but the scale factor is too small to allow proper storage of the result of the addition, leading to saturation.

To repeat the argument presented in Shukla et al. (2017), Figure 3 illustrates the importance of selecting a correct scaling factor to represent multiple values in an input vector or an input matrix. Three values are given as inputs to TrueNorth (2, 4, and 5) and represented as spike trains. In Figure 3A, all values have been scaled by the maximum-magnitude element 5, so all values can be represented within the available range of spiking rate. In Figure 3B, the inputs are scaled by a value smaller than the maximum magnitude, so saturation occurs: two elements (4 and 5) are represented by the same spike rate. Selecting the correct scaling factor is important when spike based arithmetic operations may produce results that are larger than any of the inputs. Figure 3C shows addition between two values represented as spike trains. Although both operands can be represented exactly with a scale factor of 4, the result of the addition is greater than the chosen scale factor, so the representation saturates and the result is inaccurate.

In implementating algorithms on TrueNorth, therefore, we must choose a scale factor that ensures that the intermediate computations never saturate. On the other hand, the scale factor should not be much larger than necessary, as this will result in loss of precision for the spike-train representations.

### 2.2. Range analysis to determine input scaling factor

A Hopfield linear solver (Lendaris et al., 1999; Shukla et al., 2017) can be used to compute the Moore-Penrose generalized matrix inverse based on the mathematical principles proposed by Ben-Israel and Charnes (1963). This section derives scaling factors that must be applied to the inputs to the system to guarantee that the vectors *X*_*k*_ that arise in the stationary iterative process Equation (4) [equivalently, Equation 8] have no elements greater than 1 in absolute value, for all *k*. This requirement is achieved by means of a scaling factor η applied to the right-hand side *B* in Equation (1).

For purposes of this section we define the max-norm of a matrix to be its largest element in absolute value, that is,

(10) $$\begin{array}{l} \|Y{\|}_{\max}:=\underset{i,j}{\max}|{\left[Y\right]}_{ij}|. \end{array}$$

(Note that when *Y* is a vector, the max-norm is the same as the ∞-norm.) Suppose that the (*i, j*) element of *Y* is the one that achieves the maximum norm. We have that

$$\|Y{\|}_{2}\geq \frac{\|Ye_{j}{\|}_{2}}{\|e_{j}{\|}_{2}}\geq |{\left[Y\right]}_{ij}|,\text{ for any }i\text{,}$$

where *e*_*j*_ is the vector whose elements are all zero except for a 1 in position *j*. Thus

(11) $$\begin{array}{l} \|Y{\|}_{2}\geq \|Y{\|}_{\max}. \end{array}$$

We write the singular value decomposition of *A* as follows:

(12) $$\begin{array}{l} A=U\Sigma V^{T}, \end{array}$$

where *U* is an *M* × *N* matrix with orthornormal columns, Σ is an *N* × *N* diagonal matrix with nonnegative diagonals, and *V* is an *N* × *N* orthogonal matrix. In fact, the diagonals of Σ are the singular values of *A*:

(13) $$\begin{array}{l} \Sigma =\text{diag}\left(\sigma_{1},\sigma_{2},\ldots ,\sigma_{N}\right), \end{array}$$

where σ_1_ ≥ σ_2_ ≥ … ≥ σ_*N*_ ≥ 0. We further use notation

(14) $$\begin{array}{l} \sigma_{\max}:=\sigma_{1},\;\sigma_{\min}:=\underset{\sigma_{i}>0}{\min}\sigma_{i}. \end{array}$$

In this notation, we have that $\lambda_{\max}\left(A^{T}A\right)=\sigma_{\max}^{2}$, so that condition Equation (7) becomes

(15) $$\begin{array}{l} 0<\alpha <\frac{2}{\sigma_{\max}^{2}}. \end{array}$$

Note too that $\|A{\|}_{2}=\|A^{T}{\|}_{2}=\sigma_{\max}$.

The following claim shows how we can scale the elements of *B* to ensure that ‖*X*_*k*_‖_max_ ≤ 1 for all *k*.

CLAIM 1. *For the iterative process defined by Equation (4), and supposing that condition Equation (15) holds, we have that*

(16) $$\begin{array}{l} \|X_{l}{\|}_{\max}\leq \frac{2}{\sigma_{\min}}\sqrt{MN}\|B{\|}_{\max},\;forl=0,1,2,\ldots . \end{array}$$

*Proof*: By applying Equation (4) recursively, we have for all *l* that

(17) $$\begin{array}{l} X_{l}=\alpha \overset{l}{\underset{k=0}{\sum }}{\left(I-\alpha A^{T}A\right)}^{k}A^{T}B. \end{array}$$

From Equation (12) we have that, *I* − α*A*^*T*^*A* = *V*(*I*−αΣ^2^)*V*^*T*^, so that, $X_{l}=\alpha \overset{l}{\underset{k\;=\;0}{\sum }}V{\left(I-\alpha \Sigma^{2}\right)}^{k}\Sigma U^{T}B$. By multiplying both sides by *V*^*T*^, we have that

$$\begin{array}{l} V^{T}X_{l}=\alpha {\left[\overset{l}{\underset{k=0}{\sum }}{\left(I-\alpha \sigma_{i}^{2}\right)}^{k}\sigma_{i}U_{\cdot i}^{T}B\right]}_{i=1,2,\ldots ,N}, \end{array}$$

where *U*_·*i*_ is the *i*th column of *U*. By carrying out the summation, we have

$${\left[V^{T}X_{l}\right]}_{i\cdot }=\alpha \frac{1-{\left(1-\alpha \sigma_{i}^{2}\right)}^{l+1}}{1-\left(1-\alpha \sigma_{i}^{2}\right)}\sigma_{i}U_{\cdot i}^{T}B,\;i=1,2,\ldots ,N,$$

so that

$$\begin{array}{l} {\left[V^{T}X_{l}\right]}_{i\cdot }=\{\begin{array}{ll} 0 & \text{for }\sigma_{i}=0;\\ \frac{1}{\sigma_{i}}\left[1-{\left(1-\alpha \sigma_{i}^{2}\right)}^{l+1}\right]U_{\cdot i}^{T}B & \text{for }\sigma_{i}>0. \end{array} \end{array}$$

Since 1 − α$\sigma_{i}^{2}$ ∈ (−1, 1) for all *i* = 1,2*,…,N* with σ_*i*_ > 0, we have for such *i* that

(18) $$\begin{array}{l} \|{\left[V^{T}X_{l}\right]}_{i\cdot }{\|}_{\max}\leq \frac{2}{\sigma_{i}}\|U_{\cdot i}^{T}B{\|}_{\max}\leq \frac{2}{\sigma_{i}}\|U_{\cdot i}{\|}_{1}\|B{\|}_{\max}\\ \;\leq \frac{2}{\sigma_{\min}}\sqrt{M}\|B{\|}_{\max} \end{array}$$

where the last inequality follows from ‖*U*_·*i*_‖_2_ = 1, the standard inequality that relates ‖·‖_2_ to ‖·‖_1_, and the definition (14). By considering *V*^*T*^*X*_*l*_ one column at a time, we have

$$\begin{array}{l} \|X_{l}{\|}_{\max}=\|VV^{T}X_{l}{\|}_{\max}\leq \|V{\|}_{1}\|V^{T}X_{l}{\|}_{\max}\\ \;\leq \sqrt{N}\frac{2}{\sigma_{\min}}\sqrt{M}\|B{\|}_{\max}, \end{array}$$

and by applying Equation (14), we obtain the result.        □

An immediate corollary of this result is that if we re place *B* by *B*/(η‖*B*‖_max_) in Equation (1), where

(19) $$\begin{array}{l} \eta :=\frac{2}{\sigma_{\min}}\sqrt{MN}, \end{array}$$

then the matrices *X*_*l*_ produced by the iterative process Equation (8) have ‖*X*_*l*_‖_max_ ≤ 1 for all *l* = 0, 1, 2, …. We note too that from the definition Equation (9b) of *W*_ff_ and Equation (19), we have by setting *B* = *I* and *l* = 0 in Claim 1 that

(20) $$\begin{array}{l} \|W_{\text{ff}}\|/\eta \leq 1. \end{array}$$

By applying this scaling, and writing the solution *X* of Equation (1) as an infinite sum, we have

(21) $$\begin{array}{l} X=\left(\eta {\left\|B\right\|}_{\max}\right)\overset{\infty }{\underset{k=0}{\sum }}{\left(I-\alpha A^{T}A\right)}^{k}\alpha A^{T}\frac{B}{\eta {\left\|B\right\|}_{\max}}\\ \;=\left(\eta {\left\|B\right\|}_{\max}\right)\overset{\infty }{\underset{k=0}{\sum }}{\left(I-\alpha A^{T}A\right)}^{k}\alpha A^{T}B_{n}, \end{array}$$

where *B*_*n*_: = *B*/(η‖*B*‖_max_). We use *H*_*j*_ to denote the *j*th scaled partial summation in Equation (21), that is

(22a) $$\begin{array}{l} H_{j+1}=\sum_{k=0}^{j}{\left(W_{\text{hop}}\right)}^{k}W_{\text{ff}}\frac{B}{\eta {\left\|B\right\|}_{\max}} \end{array}$$

(22b) $$\begin{array}{l} =\sum_{k=0}^{j}{\left(W_{\text{hop}}\right)}^{k}W_{\text{ff}}B_{n} \end{array}$$

(22c) $$\begin{array}{l} =W_{\text{hop}}H_{j}+W_{\text{ff}}B_{n}. \end{array}$$

By setting *j* = 0 in Equation (22c), we obtain

(23) $$\begin{array}{l} H_{1}=W_{\text{ff}}B_{n}. \end{array}$$

Note that *H*_*l*_ and *X*_*l*_ differ from each other only by the scaling factor η‖*B*‖_max_, so we have from Claim 1 and Equation (19) that

(24) $$\begin{array}{l} \|H_{l}{\|}_{\max}=\frac{1}{\eta \|B{\|}_{\max}}\|X_{l}{\|}_{\max}\leq 1,\;l=1,2,\ldots , \end{array}$$

and thus

(25) $$\begin{array}{l} \|H_{l}{\|}_{2}\leq \sqrt{NP},\;l=1,2,\ldots . \end{array}$$

### 2.3. Weight assignment

We present two techniques to encode weights for the Hopfield neural network based linear solver of section 2.1. In the first subsection, we consider hardcoding the Hopfield neural network weights as TrueNorth neuron parameters. The extracted features of the image are used to compute weight matrices *W*_hop_ and *W*_ff_, and these are converted further as TrueNorth neuron weight and threshold parameters. This scheme is suitable when the initial features do not change, as in 2-D image tracking. In the second subsection, we see how the computations are performed when weights are introduced as spikes. This scheme is appropriate for scenarios in which the initial conditions may vary frequently, such as optical flow and inverse kinematics.

#### 2.3.1. Hopfield neural network features encoded as TrueNorth weights and threshold

**Figure 4:**
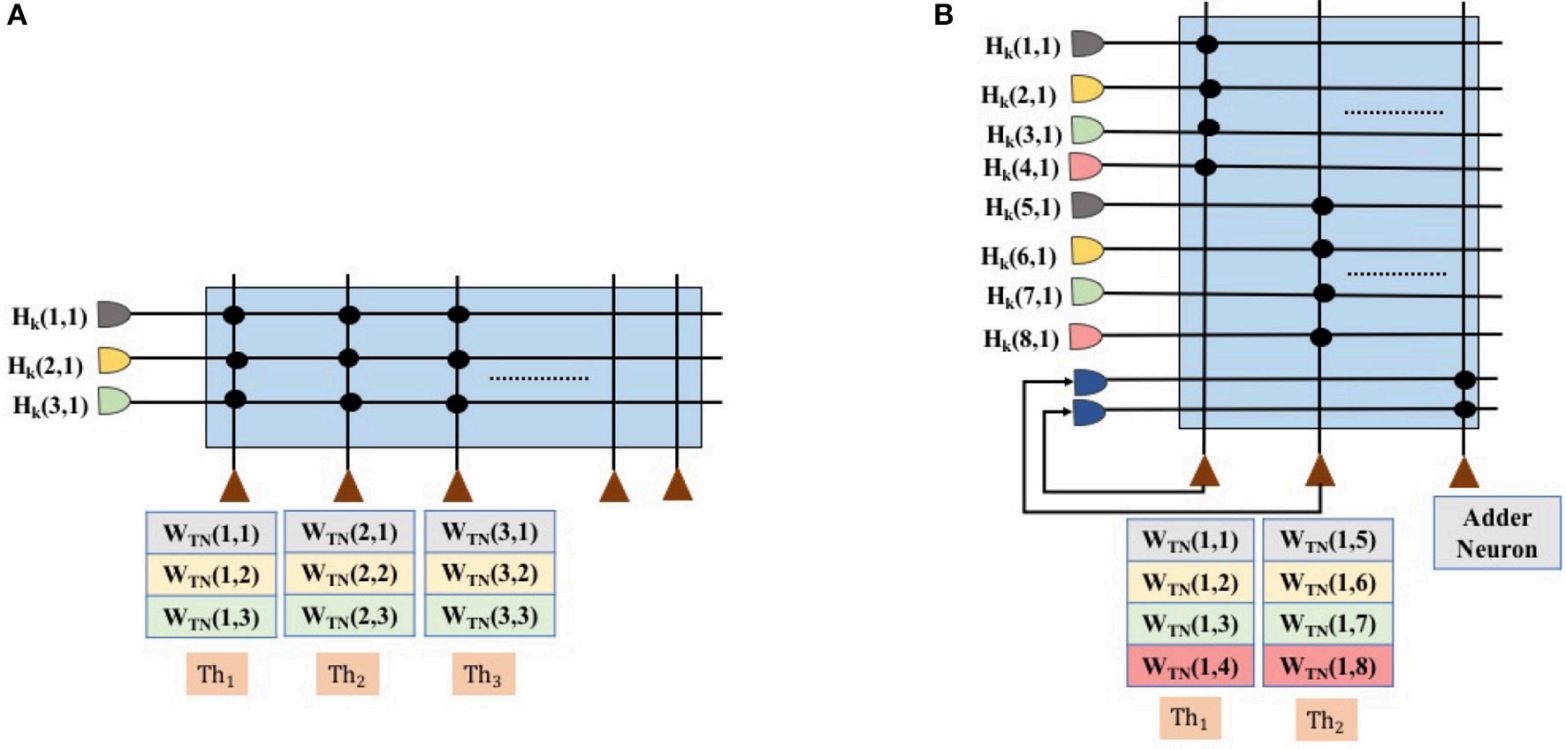
Synapse connection showing the dot product between first column of *H*_*k*_ and the weight matrix *W*_hop_, and the corresponding threshold values for each neuron. **(A)** Shows the matrix dot product for the scenario in which *W*_ff_ and *W*_hop_ can be encoded using a single neuron. **(B)** Shows the matrix dot product for the scenario where *W*_ff_ and *W*_hop_ cannot be encoded using a single neuron. We would need multiple neurons to compute partial sums and later add them up together.

To perform matrix multiplication with weight matrices *W*_ff_ and *W*_hop_, the floating point values of these two weight matrices are encoded as a ratio of TrueNorth weights to thresholds; see Algorithm 1. Here, a single synapse is used for each term in the dot product computation. In TrueNorth, each neuron can have up to four axon types as input, each of which can be assigned a unique synaptic weight in the range [−255, 255]. Figure 4A shows the synaptic connections in TrueNorth that implement dot product between the vector [*H*_*k*_(1, 1);*H*_*k*_(2, 1);*H*_*k*_(3, 1)] and the columns of the 3 × 3 weight matrix *W*_hop_ (which can have either positive or negative values). Each of the three values in *H*_*k*_ have been assigned a different axon type, so that they are multiplied with a corresponding weight value to compute a dot product of the form *w*_1_*H*_*k*_(1, 1) + *w*_2_*H*_*k*_(2, 1) + *w*_3_*H*_*k*_(3, 1). Each neuron *i*, has its reset mode set to linear reset (γ_*i*_ = 1) and rest of the parameters of the LIF neuron have the default initial value.

**Algorithm 1:** Computes the weights and threshold values for performing dot product on TrueNorth

**Input:** Floating point values in the *i*^*th*^ row of weight matrices (*W*_*i*, ._)
**Output:** Assigned TrueNorth weight and threshold
1: **procedure** WeightThresholdAssignment
2: $\text{Threshold}=\text{Round}\left(\frac{255}{\underset{i}{\max}\left(\left|W_{i,.}\right|\right)}\right)$
3: $\text{Weights}=\text{Round}\left(\frac{255}{\underset{i}{\max}\left(\left|W_{i,.}\right|\right)}\times W_{i,.}\right)$
4: **end procedure**

**Figure 5:**
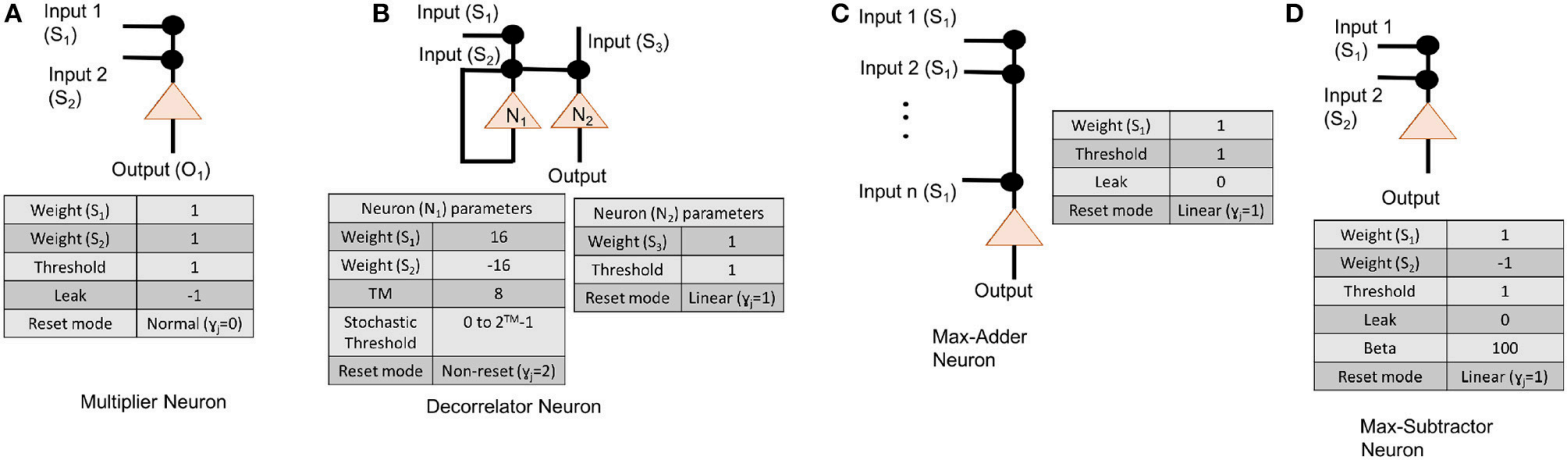
Neuron parameters for **(A)** multiplier neuron that is modeled as AND gate, **(B)** an subtractor neuron that is meant to implement max-sub function, **(C)** an adder neuron, and **(D)** decorrelator neurons.

Figure 4A presents the scenario where all weights in the Hopfield neural network (both *W*_ff_ and *W*_hop_) can be encoded on a single TrueNorth neuron. Using all of the four axon types available in a single TrueNorth neuron, we can encode a Hopfield neural network that has four *W*_ff_ and *W*_hop_ neurons. For scenarios where the Hopfield neural network might have more than four neurons in either *W*_ff_ or *W*_hop_, then the matrix multiplication would have to be divided as partial sums across multiple TrueNorth neurons. Figure 4B presents the setup for multiplying vector *H*_*k*_ by a single column of the matrix *W*_hop_. Multiple neurons would be required to handle partial sums in matrix multiplication. Partial summation of matrix dot product are computed in neurons *N*_1_ and *N*_2_. Both of these neurons have linear reset mode, and their weight and threshold values are computed using Algorithm 1. Once the partial sums have been computed, the results would go through a separate adder neuron where all of the intermediate sums would be computed. LIF neuron parameters for an adder neuron is shown in Figure 5C.

#### 2.3.2. Hopfield neural network features using spiking inputs

For applications such as optical flow and inverse kinematics, where the initial input conditions may change dynamically, the hard-coding of TrueNorth weights discussed in previous subsection is not appropriate. We need an algorithm in which TrueNorth neurons can be used as arithmetic computation units and operate over spiking inputs. Cassidy et al. (2013) show that when the data is represented as stochastic rate-based coding, the theory of stochastic computing (as presented in the survey paper of Alaghi and Hayes, 2013) shows that neurons can perform such arithmetic operations as multiplication, addition, subtraction, and division. Algorithm 2 shows the computation scheme for representing the Hopfield neural network weight matrices *W*_hop_ and *W*_ff_ using spikes. Figure 5A,C,D shows the LIF parameters of TrueNorth neurons that needs to be set to perform arithmetic operations such as multiplication, addition and subtraction, respectively.

**Algorithm 2:** Computes weight matrices *W*_ff_ and *W*_hop_ using spiking inputs

**Input:** Spiking coding of the elements in the matrices $\frac{I}{2}$, $\left(\sqrt{\frac{\alpha }{2}}\right)A^{T}$, $\left(\sqrt{\frac{\alpha }{2}}\right)A$, $\frac{\alpha A^{T}}{\eta }$. Before giving these elements as input to Truenorth, separate the elements into positive and negative domains.
**Output:** Assigned TrueNorth weight and threshold
1: **procedure** ComputeWeightsUsingSpikes
2: ${\left(\alpha A^{T}A\right)}^{+}=\max\left(\alpha A^{T}A,0\right);$
3: ${\left(\alpha A^{T}A\right)}^{-}=\max\left(-\alpha A^{T}A,0\right);$
4: ${\left(\alpha A^{T}\right)}^{+}=\max\left(\alpha A^{T},0\right);$
5: ${\left(\alpha A^{T}\right)}^{-}=\max\left(-\alpha A^{T},0\right);$
6: $P_{1}=\max\left(\frac{I}{2}-\frac{{\left(\alpha A^{T}A\right)}^{+}}{2},0\right);$
7: $P_{2}=\max\left(\frac{{\left(\alpha A^{T}A\right)}^{+}}{2}-\frac{I}{2},0\right);$
8: $P_{3}=\left(\frac{{\left(\alpha A^{T}A\right)}^{-}}{2}\right);$
9: $W_{\text{hop}}^{+}=2(P_{1}+P_{3});$
10: $W_{\text{hop}}^{-}=2(P_{2});$
11: $W_{\text{ff}}^{+}={\left(\alpha A^{T}\right)}^{+}/\eta ;$
12: $W_{\text{ff}}^{-}={\left(\alpha A^{T}\right)}^{-}/\eta ;$
13: **end procedure**

Since the matrix computations for *W*_hop_ and *W*_ff_ will be done in the hardware itself, we need to reconstruct the iteration formula in such a way that every term that serves as an input to the hardware has magnitude <1, otherwise the computations might saturate and give us the wrong result. We rewrite Equation (22a) as follows, to ensure that each bracketed term has all its elements in the range [−1, 1]:

(26) $$\begin{array}{l} H_{j+1}=\overset{j}{\underset{k=0}{\sum }}{\left(\frac{I}{2}-\left(\sqrt{\frac{\alpha }{2}}A^{T}\right)\left(\sqrt{\frac{\alpha }{2}}A\right)\right)}^{k}2^{k}\left(\frac{\alpha A^{T}}{\eta }\right)\left(\frac{B}{B_{\max}}\right). \end{array}$$

We state the formal claim as follows.

CLAIM 2. *All elements of the matrices* $(\sqrt{\frac{\alpha }{2}}A)$, $(\sqrt{\frac{\alpha }{2}}A^{T})$, *W*_hop_, *and* $(\frac{\alpha A^{T}}{\eta })$ *lie in the interval* [−1, 1]. *That is, the max-norms Equation (10) of these four matrices are all less than* 1.

Proof: Because of Equation (11), it suffices to show that ‖·‖_2_ ≤ 1 for all four of the matrices in question.

For the first matrix, note from Equation (15) that $\sqrt{\alpha /2}\leq 1/\sigma_{\max}=1/\|A{\|}_{2}$. Thus $\|\sqrt{\frac{\alpha }{2}}A{\|}_{2}\leq 1$, as required. The proof for the second matrix is identical.

For the third matrix we note that *W*_hop_ is a square symmetric matrix with eigenvalues in the range [−1, 1]. Thus the eigenvalues of $W_{\text{hop}}^{\text{2}}$ will be in the range [0, 1], so ‖*W*_hop_‖_2_ ≤ 1, as required.

For the fourth matrix, we have from Equation (15), the definition of η in Equation (19), and the fact that ‖*A*‖_2_ = σ_max_ that

$$\|\frac{\alpha A^{T}}{\eta }{\|}_{2}\leq \frac{2}{\sigma_{\max}^{2}}\sigma_{\max}\frac{\sigma_{\min}}{2\sqrt{MN}}=\frac{\sigma_{\min}}{\sigma_{\max}\sqrt{MN}}\leq 1.$$

        ■

### 2.4. Implementation

The spiking neural substrates can operate only for values in the range [0, 1]. Thus, to perform computation on numbers that can be either positive or negative, the computations must be divided into two separate domains, one working with the positive parts of the matrices and one with the negative parts. Algorithms 3 and 4 implement the formula Equation (22b). Algorithm 3 performs the preprocessing step or the feedforward path of the neural network architecture, while Algorithm 4 implements the recurrent part of the Hopfield architecture. The steps shown in Algorithms 3 and 4 ensure that the intermediate computation values never saturate. This is managed by performing subtraction of intermediate results followed by addition in the final step. Since the input values were normalized by the scaling factor, as shown in Equation (19), the addition of partial sums would never saturate. We use the following definition of the positive and negative parts of a matrix:

(27) $$\begin{array}{l} Y^{+}:=\max\left(Y,0\right),\;Y^{-}:=\max\left(-Y,0\right), \end{array}$$

where the max-operation is applied component-wise. The proposed architecture ensures that nonzero elements in the positive-part matrices have zeros in the corresponding elements of the negative-part matrices, and vice versa. We do not have to scale the values in Algorithm 4 while computing matrices *M*, *PS*_1_, and *PS*_2_ because Claim 1 guarantees that no quantity will exceed 1, by choice of scale factor η. The max function used in Algorithms 2, 3, and 4 can be implemented with a LLIF (linear leaky integrated fire) neuron.

**Algorithm 3:** Computes the scaled value of input features *B* based on *W*_ff_ and the normalizing factor. These scaled values serve as the input for recurrent network

**Input:** Coordinates of the current input features *B*
**Output:** Scaled values of input features for the recurrent network. These values are divided among four domains
1: **procedure** Preprocessing
2: **if** Weights are hard-coded on TrueNorth **then**
3: *B*_*n*_ = Normalize(*B*, η ‖*B*‖_max_);
4: **else if** Weights are given as spiking inputs **then**
5: *B*_*n*_ = Normalize(*B*, ‖*B*‖_max_);
6: **end if**
7: $T^{\langle +,+\rangle }=\max\left(W{\text{ff}}^{+}B_{n}^{+}-W{\text{ff}}^{+}B_{n}^{-},0\right)$;
8: $T^{\langle +,-\rangle }=\max\left(W{\text{ff}}^{+}B_{n}^{-}-W{\text{ff}}^{+}B_{n}^{+},0\right)$;
9: $T^{\langle -,-\rangle }=\max\left(W{\text{ff}}^{-}B_{n}^{-}-W{\text{ff}}^{-}B_{n}^{+},0\right)$;
10: $T^{\langle -,+\rangle }=\max\left(W{\text{ff}}^{-}B_{n}^{+}-W{\text{ff}}^{-}B_{n}^{-},0\right)$;
11: $B_{s}^{\langle +,+\rangle }=\max\left(T^{\langle +,+\rangle }-T^{\langle -,+\rangle },0\right)$;
12: $B_{s}^{\langle -,+\rangle }=\max\left(T^{\langle -,+\rangle }-T^{\langle +,+\rangle },0\right)$;
13: $B_{s}^{\langle -,-\rangle }=\max\left(T^{\langle -,-\rangle }-T^{\langle +,-\rangle },0\right)$;
14: $B_{s}^{\langle +,-\rangle }=\max\left(T^{\langle +,-\rangle }-T^{\langle -,-\rangle },0\right)$;
15: **end procedure**

**Algorithm 4:** Solve for system of linear equations defined as *AX* = *B*. Computations are divided into negative and positive parts

**Input:** Matrices *A* and *B* that have been divided into positive and negative domains
**Output:** Solution for the system of linear equation, matrix *X*
1: **procedure** HopfieldSolver_Split
2: **while** δ ≥ Minimum Error **do**
3: $M^{\langle +,+\rangle }=\left(B_{s}^{\langle +,+\rangle }\right)+\left(W{\text{hop}}^{+}H_{k}^{+}\right)$;
4: $M^{\langle +,-\rangle }=\left(B_{s}^{\langle +,-\rangle }\right)+\left(W{\text{hop}}^{+}H_{k}^{-}\right)$;
5: $M^{\langle -,-\rangle }=\left(B_{s}^{\langle -,-\rangle }\right)+\left(W{\text{hop}}^{-}H_{k}^{-}\right)$;
6: $M^{\langle -,+\rangle }=\left(B_{s}^{\langle -,+\rangle }\right)+\left(W{\text{hop}}^{-}H_{k}^{+}\right)$;
7: $PS_{1}^{\langle +,+\rangle }=\max\left(M^{\langle +,+\rangle }-M^{\langle +,-\rangle },0\right)$;
8: $PS_{1}^{\langle +,-\rangle }=\max\left(M^{\langle +,-\rangle }-M^{\langle +,+\rangle },0\right)$;
9: $PS_{1}^{\langle -,-\rangle }=\max\left(M^{\langle -,-\rangle }-M^{\langle -,+\rangle },0\right)$;
10: $PS_{1}^{\langle -,+\rangle }=\max\left(M^{\langle -,+\rangle }-M^{\langle -,-\rangle },0\right)$;
11: $PS_{2}^{\langle +,+\rangle }=\max\left(P\overset{\langle +,+\rangle }{\underset{1}{S}}-PS_{1}^{\langle -,+\rangle },0\right)$;
12: $PS_{2}^{\langle -,+\rangle }=\max\left(P\overset{\langle -,+\rangle }{\underset{1}{S}}-PS_{1}^{\langle +,+\rangle },0\right)$;
13: $PS_{2}^{\langle -,-\rangle }=\max\left(P\overset{\langle -,-\rangle }{\underset{1}{S}}-PS_{1}^{\langle +,-\rangle },0\right)$;
14: $PS_{2}^{\langle +,-\rangle }=\max\left(P\overset{\langle +,-\rangle }{\underset{1}{S}}-PS_{1}^{\langle -,-\rangle },0\right)$;
15: ${\tilde{\text{H}}}_{k+1}^{+}=\left(PS_{2}^{\langle +,+\rangle }+PS_{2}^{\langle -,-\rangle }\right)$;
16: ${\tilde{\text{H}}}_{k+1}^{-}=\left(PS_{2}^{\langle +,-\rangle }+PS_{2}^{\langle -,+\rangle }\right)$;
17: **if** Weights are hard-coded on TrueNorth **then**
18: $H_{k+1}^{+}={\tilde{\text{H}}}_{k+1}^{+}$;
19: $H_{k+1}^{-}={\tilde{\text{H}}}_{k+1}^{-}$;
20: **else if** Weights are given as spiking inputs **then**
21: $H_{k+1}^{+}=\text{Decorrelate}\left({\tilde{\text{H}}}_{k+1}^{+}\right)$;
22: $H_{k+1}^{-}=\text{Decorrelate}\left({\tilde{\text{H}}}_{k+1}^{-}\right)$;
23: **end if**
24: $\delta =\left(H_{k+1}^{+}-H_{k+1}^{-}\right)-\left(H_{k}^{+}-H_{k}^{-}\right)$;
25: *k* = *k*+1;
26: **end while**
27: *X*_*k*_ = Rescale(*H*_*k*_, η ‖*B*‖_max_);
28: **end procedure**

To implement these arithmetic operations we set the TrueNorth neuron parameters appropriately. A detailed description of individual neuron parameters and their behavior with respect to TrueNorth's spiking neurons can be found in Cassidy et al. (2013). Figure 5C shows the neuron parameters and connections for implementing an adder function that is required to compute variables such as *M* in Algorithm 4. Similarly, Figure 5B shows the neuron parameters and connections for max-subtractor neuron that is meant to compute variables such as *PS*_1_, and *PS*_2_ in Algorithm 4, or, variable *B*_*s*_ in Algorithm 3.

#### 2.4.1. Computation with spiking weights

For applications in which matrix *A* might change dynamically, it is not possible to hard-code the weights on TrueNorth. Instead, we borrow concepts from stochastic computing (Gaines, 1967; Alaghi and Hayes, 2013) to perform multiplication between input streams using a single neuron. In stochastic computing, if the inputs are represented as independent streams of bits, then the multiplication between these two values can be implemented with just one AND gate. In our implementation, the values are represented as stochastically rate coded spikes, similar to bit streams mentioned earlier. The AND gate can be modeled with an LLIF neuron as shown in Figure 5A.

Since the computation involves sending the values through a recurrent path, it is crucial to maintain independence of spike occurrence between the inputs from feedforward path and inputs from recurrent path. Therefore, the inputs that are fed back need to be passed through a decorrelator, as shown in Figure 1. The decorrelator (presented in Chen and Hayes, 2014) preserves the spiking rate of the input signal, but makes the occurrence of spikes independent of the randomly generated feedforward values. This is done by incrementing the membrane potential when it receives a spike, let the neuron firing threshold vary stochastically and once the neuron fires decrease the membrane potential by the same magnitude as it was increased. Parameters for modeling a decorrelator using TrueNorth neurons are shown in Figure 5D.

### 2.5. Precision

Computations on the neural network substrate generally have limited precision, producing accumulated error in the output. The two main sources of computation error are (1) quantization of the weights and the input, and (2) stochastic computations, when computations are performed using spiking weights. In this section, we first find the upper bound for the output error for the case where weights are hard-coded into the neural network substrate, considering only quantization errors in the weights and the input. We then update the upper bound for the case in which weights are represented using spikes, so that further stochastic errors arise in the computations.

#### 2.5.1. Quantization error

Given that the elements in the input and weight matrices contain quantization errors, we examine quantization errors in the output. We denote the errors in *W*_hop_, *W*_ff_, and *B*_*n*_ by Δ*W*_hop_, Δ*W*_ff_, and Δ*B*_*n*_, respectively. If δ_hop_, δ_ff_, and δ_bn_ represent upper bounds on the individual elements of Δ*W*_hop_, Δ*W*_ff_, and Δ*B*_*n*_, respectively, we have by the dimensions of *A* (*M* × *N*) and *B* (*M* × *P*) that

(28a) $$\begin{array}{l} \|\Delta W_{\text{hop}}{\|}_{2}\leq \|\Delta W_{\text{hop}}{\|}_{F}\leq N\delta_{\text{hop}}, \end{array}$$

(28b) $$\begin{array}{l} \|\Delta W_{\text{ff}}\|\leq \sqrt{NM}\delta_{\text{ff}}, \end{array}$$

(28c) $$\begin{array}{l} \|\Delta B_{n}\|\leq \sqrt{MP}\delta_{\text{bn}}. \end{array}$$

These errors produce an error Δ*H* in the final output matrix *H* resulting from iterative application of the formula Equation (22c) (or its equivalents). Assuming we know the exact values of the input matrices *A* and *B*_*n*_ and weight matrices *W*_ff_ and *W*_hop_ without quantization, we can find an upper bound on the norm of the output error Δ*H*^*Q*^. This result requires a condition on the singular values of the modified iteration matrix *W*_hop_ + Δ*W*_hop_, without which the output errors Δ*H*_*j*_ at successive iterations *j* may diverge. We define

(29) $$\begin{array}{l} {\bar{\sigma }}_{\max}:=\sigma_{\max}\left(W_{\text{hop}}+\Delta W_{\text{hop}}\right), \end{array}$$

and require the following condition to hold:

(30) $$\begin{array}{l} {\bar{\sigma }}_{\max}<1. \end{array}$$

By using the definition Equation (9a), together with Equations (12), (13), (28), and the Wielandt-Hoffmann inequality, we have

(31) $$\begin{array}{l} {\bar{\sigma }}_{\max}\leq \sigma_{\max}\left(W_{\text{hop}}\right)+\|\Delta W_{\text{hop}}{\|}_{F}\leq \max\left(\left|1-\alpha \overset{2}{\underset{1}{\sigma }}|,|1-\alpha \sigma_{N}^{2}\right|\right)\\ +N\delta_{\text{hop}}. \end{array}$$

Thus a sufficient condition for Equation (30) is

(32) $$\begin{array}{l} \max\left(\left|1-\alpha \overset{2}{\underset{1}{\sigma }}|,|1-\alpha \sigma_{N}^{2}\right|\right)+N\delta_{\text{hop}}<1. \end{array}$$

Note that this condition can be satisfied *only if A has full rank*, that is, σ_*N*_ > 0. If we assume that in addition to Equation (15), α also satisfies the (not very restrictive) condition

(33) $$\begin{array}{l} 0<\alpha <\frac{1}{\sigma_{N}^{2}}, \end{array}$$

then $|1-\alpha \sigma_{N}^{2}|=1-\alpha \sigma_{N}^{2}>0$, and Equation (32) can hold only if $1-\alpha \sigma_{N}^{2}+N\delta_{\text{hop}}<1$, that is,

(34) $$\begin{array}{l} \delta_{\text{hop}}<\frac{1}{N}\alpha \sigma_{N}^{2}. \end{array}$$

This condition bounds the allowable error in the elements of *W*_hop_ in terms of the steplength α and the spectrum of *A*.

Recall for the following result that the dimensions of matrices *A* and *B*_*n*_ are *M* × *N* and *M* × *P*, respectively.

CLAIM 3. *Suppose that Equation (30) holds. Then the upper bound on 2-norm of accumulated error is as follows:*

$$\|\Delta H^{Q}{\|}_{2}\leq \frac{1}{\left(1-{\bar{\sigma }}_{\max}\right)}E_{Q},$$

where E_Q_ is defined by

(35) $$\begin{array}{l} E_{Q}:=(\delta \text{ff}\sqrt{NM}∥B_{n}∥+∥W_{\text{ff}}∥\delta \text{bn}\sqrt{MP}+\delta \text{ff}\delta \text{bn}\sqrt{NM}\sqrt{MP}\\ +\delta_{\text{hop}}N\sqrt{NP}). \end{array}$$

Proof: We rewrite Equation (22c) to include the errors in the constituent quantities:

(36) $$\begin{array}{l} H_{k}+\Delta H_{k}^{Q}=\left(W_{\text{ff}}+\Delta W_{\text{ff}}\right)\left(B_{n}+\Delta B_{n}\right)+\left(W_{\text{hop}}+\Delta W_{\text{hop}}\right)\\ \left(H_{k-1}+\Delta H_{k-1}^{Q}\right). \end{array}$$

By subtracting *H*_*k*_ from both sides of Equation (36), we obtain

(37a) $$\begin{array}{l} \Delta H_{k}^{Q}=\Delta H_{1}^{Q}+\Delta W_{\text{hop}}H_{k-1}+\left(W_{\text{hop}}+\Delta W_{\text{hop}}\right)\Delta H_{k-1}^{Q},\\ k=2,3,\ldots \end{array}$$

(37b) $$\begin{array}{l} \Delta H_{1}^{Q}=\Delta W_{\text{ff}}B_{n}+W_{\text{ff}}\Delta B_{n}+\Delta W_{\text{ff}}\Delta B_{n}. \end{array}$$

By taking norms in Equation (37a) and applying standard norm inequalities, we obtain

(38) $$\begin{array}{l} \|\Delta H_{k}^{Q}{\|}_{2}\leq \|\Delta H_{1}^{Q}{\|}_{2}+\|\Delta W_{\text{hop}}{\|}_{2}\|H_{k-1}^{Q}{\|}_{2}+\|W_{\text{hop}}\\ +\Delta W_{\text{hop}}{\|}_{2}\|\Delta H_{k-1}^{Q}{\|}_{2}\\ \leq \|\Delta H_{1}^{Q}{\|}_{2}+N^{3/2}P^{1/2}\delta_{\text{hop}}+{\bar{\sigma }}_{\max}\|\Delta H_{k-1}^{Q}{\|}_{2}, \end{array}$$

where we used Equations (25) and (28) to bound the second term. By applying this formula recursively for *k* − 1, *k* − 2, …, 1, we obtain

(39) $$\begin{array}{l} \|\Delta H_{k}^{Q}{\|}_{2}\leq \overset{k-1}{\underset{l=0}{\sum }}{\bar{\sigma }}_{\max}^{l}\left(\|\Delta H_{1}^{Q}{\|}_{2}+N^{3/2}P^{1/2}\delta_{\text{hop}}\right)\\ \leq \frac{1}{1-{\bar{\sigma }}_{\max}}\left(\|\Delta H_{1}^{Q}{\|}_{2}+N^{3/2}P^{1/2}\delta_{\text{hop}}\right). \end{array}$$

We now obtain a bound on $\|\Delta H_{1}^{Q}{\|}_{2}$. By taking norms in Equation (37b) and using Equation (28), we obtain

$$\begin{array}{l} \|\Delta H_{1}^{Q}{\|}_{2}\leq \|\Delta W_{\text{ff}}{\|}_{2}\|B_{n}{\|}_{2}+\|W_{\text{ff}}{\|}_{2}\|\Delta B_{n}{\|}_{2}+\|\Delta W_{\text{ff}}{\|}_{2}\|\Delta B_{n}{\|}_{2}\\ \leq \sqrt{MN}\delta \text{ff}\|B_{n}{\|}_{2}+\|W_{\text{ff}}{\|}_{2}\sqrt{MP}\delta \text{bn}+\sqrt{MN}\delta \text{ff}\sqrt{MP}\delta \text{bn}, \end{array}$$

giving the result.         ■

This claim can be used to find the amount of resources needed to generate an output *H* + Δ*H*^*Q*^ in which the error satisfies a specified bound, for example, $\|\Delta H^{Q}{\|}_{2}\leq \epsilon$. Note that the values of δ_hop_, δ_ff_, and δ_bn_ can be manipulated in various ways to meet these goals. For instance, in a neural substrate like TrueNorth, δ_bn_ can be reduced by increasing the number of time ticks at the cost of increased execution time. On the other hand, reductions in δ_hop_ can be achieved by using multiple neurosynaptic cores at the cost of increased area and power. The validation of the model is discussed in section 3. We leave exploration of such optimizations to future work.

#### 2.5.2. Stochastic error

As mentioned earlier, stochastic computation is the second key source of error in the Hopfield network. Computation in the stochastic domain is performed not on the exact values of inputs and outputs, but on their expected values. However, the random errors present in the inputs to the computations performed in the network lead to random errors in the output, which accumulate during execution of Hopfield network. In this section, we seeks bounds on stochastic error. Claim 4 shows a bound on the output error for the entire computation in terms of error bounds for a single stochastic matrix multiplication. We complement this claim by estimating the bound on stochastic error in a single stochastic matrix multiplication. It is important to note that there are no useful error bounds that hold with absolute certainty! It is possible—though highly unlikely under reasonable assumptions—for stochastic errors to overwhelm the computation. However, we can use information about the distribution of the errors to give some insight into how these errors propagate through the computation, showing conditions under which we can reasonably expect the results to be acceptably accurate.

Claim 3 defines the error bound due to quantization error, in terms of a quantity *E*_*Q*_ defined in Equation (35). We can use this definition as part of the upper bound for stochastic error. For this analysis we assume that the only stochastic computation performed in the Hopfield network is stochastic multiplication—we assume that additions are exact. Each matrix multiplication yields some stochastic error that propagates through subsequent iterations.

CLAIM 4. *Suppose that Equation (30) holds. We denote by E_M_ a bound on the stochastic multiplication error caused by multiplication of W*_ff_ *and B_n_, and denote by E_N_ a bound on stochastic multiplication error caused by multiplication of W*_hop_ *and H_k_. Let E_Q_ be defined as in Claim 3. Then the error bound for ΔH can be estimated as follows:*

$$\begin{array}{l} ∥\Delta H∥\leq \frac{1}{1-{\bar{\sigma }}_{\max}}\left(E_{Q}+E_{M}+E_{N}\right), \end{array}$$

Proof: We rewrite the Hopfield equation, introducing stochastic error terms into Equation (36), as follows:

$$\begin{array}{l} H_{k}+\Delta H_{k}=(W_{\text{ff}}+\Delta W_{\text{ff}})(B_{n}+\Delta B_{n})+\Delta_{M_{k}}+(W_{\text{hop}}\\ \;+\Delta W_{\text{hop}})(H_{k-1}+\Delta H_{k-1})+\Delta_{N_{k}}, \end{array}$$

where Δ_*M*_*k*__ and Δ_*N*_*k*__ represent the stochastic multiplication errors for *W*_ff_*B*_*n*_ and *W*_hop_*H*_*k*−1_. Our assumptions yield the following bounds on these error quantities:

(40) $$\begin{array}{l} ∥\Delta_{M_{k}}∥\leq E_{M},\;∥\Delta_{N_{k}}∥\leq E_{N}. \end{array}$$

By comparing the formula above, and denoting by $\Delta H_{k}^{Q}$ the quantization error Equation (37), we obtain the following recursive formula for total error Δ*H*_*k*_:

(41) $$\begin{array}{l} \Delta H_{k}=\Delta H_{k}^{Q}+\sum_{j=0}^{k}{\left(W_{\text{hop}}+\Delta W_{\text{hop}}\right)}^{j}\Delta_{M_{k-j}}+\sum_{j=0}^{k-1}(W_{\text{hop}}\\ \;+\Delta W_{\text{hop}}{)}^{j}\Delta_{N_{k-j}}. \end{array}$$

This formula represents a closed-form expression for the stochastic error, similar to the closed form equation that was used to derive quantization error in Equations (37a) and (37b). By taking norms, and using Equations (29) and (30) together with Equation (40), we have

$$\begin{array}{l} \left\|\Delta H_{k}\|\leq \|\Delta H_{k}^{Q}\|+\overset{k}{\underset{j=0}{\sum }}\|{\left(W_{\text{hop}}+\Delta W_{\text{hop}}\right)}^{j}\Delta_{M_{k-j}}\right\|\\ +\overset{k-1}{\underset{j=0}{\sum }}\|{\left(W_{\text{hop}}+\Delta W_{\text{hop}}\right)}^{j}\Delta_{N_{k-j}}\|\\ \leq \|\Delta H_{k}^{Q}\|+\overset{k}{\underset{j=0}{\sum }}{\bar{\sigma }}_{\max}^{j}\|\Delta_{M_{k-j}}\|+\overset{k-1}{\underset{j=0}{\sum }}{\bar{\sigma }}_{\max}^{j}\|\Delta_{N_{k-j}}\|\\ \leq \|\Delta H_{k}^{Q}\|+\frac{1}{1-{\bar{\sigma }}_{\max}}\left(E_{M}+E_{N}\right). \end{array}$$

The result now follows from the bound on $\left\|\Delta H_{k}^{Q}\right\|$ in Claim 3.        ■

We complete the error analysis by obtaining bounds *E*_*M*_ and *E*_*N*_ on the stochastic matrix multiplication error norms. Instead of finding a *certain* value for these upper bounds, we find estimates based on the distribution of the elements of the stochastic error matrices that arise in the computations. (It is possible to find a certain bound, but it is too loose to be useful, and the error that actually appears during computation rarely approaches this certain bound.)

Before describing the stochastic errors that arise from a matrix multiplication, we examine the error in multiplying two *scalars* in the range [0, 1]. Let *y*_1_ and *y*_2_ be two such scalars, and let *z* = *y*_1_*y*_2_ be their product. On the spiking substrate, *y*_1_ and *y*_2_ are represented by spike trains of length *L*, and we denote by *Y*_1_ and *Y*_2_ (respectively) their *represented* values, which are random variables. Since each spike can be thought of as a binary random variable, the expected value and variance of *Y*_1_ and *Y*_2_ are as follows:

(42a) $$\begin{array}{l} E\left(Y_{1}\right)=y_{1},\;Var\left(Y_{1}\right)=\frac{y_{1}\left(1-y_{1}\right)}{L} \end{array}$$

(42b) $$\begin{array}{l} E\left(Y_{2}\right)=y_{2},\;Var\left(Y_{2}\right)=\frac{y_{2}\left(1-y_{2}\right)}{L}. \end{array}$$

Denoting by *Z* the random variable resulting from the multiplication of *Y*_1_ and *Y*_2_, we have that *E*(*Z*) = *z* = *y*_1_*y*_2_ and

(43) $$\begin{array}{l} \sigma_{Z}^{2}=\text{Var}\left(Z\right)=\text{Var}\left(Y_{1}\right)E\left(Y_{2}\right)+\text{Var}\left(Y_{2}\right)E\left(Y_{1}\right)+\text{Var}\left(Y_{1}\right)\text{Var}\left(Y_{2}\right). \end{array}$$

The value of $\sigma_{Z}^{2}$ approaches its minimum value of 0 when *Y*_1_ and *Y*_2_ are close to 0 or 1. A closed form solution can be calculated for the *maximum* value of σ_*Z*_ over all possible *y*_1_, *y*_2_ ∈ [0, 1].

CLAIM 5. *For large L*, $\sigma_{Z}^{2}$ *reaches its maximum when* $y_{1}=y_{2}\approx \frac{2}{3}$, *and this maximum value is approximately* 0.296/*L*.

Proof: From Equations (43) and (42), we have that:

$$\begin{array}{l} \sigma_{Z}^{2}=\frac{y_{1}\left(1-y_{1}\right)y_{2}}{L}+\frac{y_{1}y_{2}\left(1-y_{2}\right)}{L}+\frac{y_{1}\left(1-y_{1}\right)y_{2}\left(1-y_{2}\right)}{L^{2}}. \end{array}$$

As *L* increases, the third term is dominated increasingly by the first two terms, so we can omit it from consideration. By taking the gradient and Hessian of the resulting approximation of $\sigma_{Z}^{2}$ with respect to (*y*_1_, *y*_2_), we obtain

$$\begin{array}{l} \text{gradient}=\frac{1}{L}\left[\begin{array}{c} y_{2}\left(1-2y_{1}\right)+y_{2}\left(1-y_{2}\right)\\ y_{1}\left(1-y_{1}\right)+y+1\left(1-2y_{2}\right) \end{array}\right],\\ \text{ Hessian}=\frac{1}{L}\left[\begin{array}{cc} -2y_{2} & 2-2y_{1}-2y_{2}\\ 2-2y_{1}-2y_{2} & -2y_{1} \end{array}\right]. \end{array}$$

It is easy to check that when *y*_1_ = *y*_2_ = 2/3, the gradient is zero and the Hessian is negative definite. Thus *y*_1_ = *y*_2_ = 2/3 is an approximate maximizer, and the value of $\sigma_{Z}^{2}$ at this point is approximately 8/(27*L*) ≈ 0.296/*L*.         ■

Note that each element of the matrix Δ_*M*_*k*__ in the proof of Claim 4 is the stochastic error that arises from taking the inner product of two vectors: one row of *W*_ff_ + Δ*W*_ff_ and one column of *B*_*n*_ + Δ*B*_*n*_. These two vectors have length *M*, and the product matrix has dimension *N* × *P*. Since we assume that no stochastic error occurs in matrix additions, the accumulated stochastic error in each element of Δ_*M*_*k*__ is the sum of *M* random variables, each with expectation 0 and variance bounded by 0.296/*L* (for large *L*). Since the variance of a sum of uncorrelated random variables is the sum of the variances, we can reasonably say that each element of Δ_*M*_*k*__ is a random variable with expectation 0 and variance bounded by 0.296*M*/*L*. An appropriate value of *E*_*M*_ in Equation (40) can be obtained by taking the Frobenius norm of an *N* × *P* matrix whose elements are all equal to the standard deviation (the square root of this variance), and multiplying by a “safety factor” greater than 1. If we choose the value 4 for this safety factor, we obtain the following value for *E*_*M*_:

$$E_{M}:=4\sqrt{NP\left(0.296M/L\right)}\approx 2.176\sqrt{MNP/L}.$$

A similar calculation involving Δ_*N*_*k*__ and *E*_*N*_, using the fact that *W*_hop_ is *N* × *N* and *H* is *N* × *P* results in the following estimate for *E*_*N*_:

$$E_{N}:=4\sqrt{NP\left(0.296N/L\right)}\approx 2.176N\sqrt{P/L}.$$

This derivation of suitable values for the bounds *E*_*M*_ and *E*_*N*_ is informal, but it suffices to give insight into how these bounds vary with the dimensions of the matrices involved, and with the length of the spike train representation *L*.

## 3. Results

This section presents the validation results and observations for the mathematical models for scaling factor and the precision analysis. For all experiments, we set α = 1.9/trace(*A*^*T*^*A*); Equation (7) guarantees convergence of the iterative process for this value of the parameter.

### 3.1. Experiments

A TrueNorth-based Hopfield linear solver was applied in the context of real-time robotics applications in Shukla et al. (2017). This article looked at three different applications—target tracking (Figure 2A), optical flow (Figure 2B), and inverse kinematics—and reported relative error and absolute error of these experiments. Each of these experiments required a different input matrix dimension, and results were reported for over 500 different input matrix values. However, Shukla et al. (2017) did not include a mathematically complete architecture, nor did it study the effects of computational limitations, both of which have been examined in this paper.

Table 1 shows the results for 15 different types of matrices, with each matrix repeated 20 times. These experiments were conducted using spike based weight representation scheme on the TrueNorth system. In total, 712 TrueNorth neurons were required to implement the proposed algorithm (just 0.067% of available hardware neurons) and we needed just 11 cores of the 4,096 available cores. In the notation of section 2.1, the dimensions are *M* = 25, *N* = 2, and *P* = 1. Thus, *A* is 25 × 2, *B* 25 × 1 and *H*_*j*_ in Equation (22a) is 2 × 1.

**Table 1:** Results for Hopfield linear solver with spike based weight representation.

**Experiment number**	**Properties of matrices *A* and *B***	**Additional comments**	**Time ticks (in millions)**	**MSE for ‖Δ*H*‖ (%)**	**SDSE for ‖Δ*H*‖ (%)**
1	Each element chosen uniformly in [−1, 1]	Most basic test for Hopfield linear solver	1.05	0.0004	0.0013
2	Each element is an integer chosen uniformly in [−100, 100]	Observe the behavior of linear solver as the input range is increased	3.5	0.0025	0.008
3	Each element chosen uniformly in [−100, 100]	Each element can have fractional values	3.5	0.0014	0.0028
4	Each element chosen uniformly in [1, 100]	All elements of *A* have the same sign	4	0.0353	0.19
5	Each element chosen uniformly in [0.001, 1]	Analyzing the convergence when the values are small	4	0.0038	0.008
6	Each element chosen uniformly in [0.0001, 1]	Analyzing the convergence when the possible values are smaller than previous experiments	4.25	0.0068	0.0234
7	Each element chosen uniformly in [−1000, 1000]	Higher precision is required for calculation	4	0.0186	0.0413
8	Each element chosen uniformly in [−10, 000, 10, 000]	Testing for the cases when even more precision is required for calculation	4	0.32	0.83
9	Each element chosen uniformly in [1, 10, 000]	Matrix *A* has elements with same sign; requires higher precision for convergence	4	1.16	2.97
10	Each element chosen uniformly in [−1000, 1000] except that 50% of elements in *A* and *B* are 0	Effect of sparsity on final result and convergence	4	0.024	0.0488
11	Each element chosen uniformly in [1, 10, 000] except for 50% zeros	Effect of sparsity on final result and convergence	4	0.24	0.94
12	Each element chosen uniformly in [0.0001, 1] except for 45% zeros in *A* and *B*	Effect of sparsity on final result and convergence when elements of *A* are small	4.25	0.0038	0.0114
13	Each element chosen uniformly in [0, 50]. For matrix *A*, ratio of smallest to largest singular values is about 0.25	Both the eigenvalues of *W*_hop_ will have magnitude close to 1, but will have opposite signs	4.25	0.37	1.01
14	Each element chosen uniformly in [−5 × 10^5^, 5 × 10^5^]	Testing for the cases when up-to 10^−6^ precision would be required for calculation	4.25	5.11	9.54
15	Each element chosen uniformly in [1, 5 × 10^5^]	Precision of better than 10^−6^ would be required for calculation and all matrix input values have the same sign	4.25	96.48	316.54

*Column 2 describes how the values of matrices A and B were generated, while Column 3 explains why these matrices were chosen. Column 4 presents the number of clock ticks (or the spike duration) for each experiment. Columns 5 and 6 show the percentage mean (MSE) and percentage standard deviation (SDSE) of the squared error of the Hopfield linear solver output relative to double-precision MATLAB quantity (‖ΔH‖)*.

In Table 1, Columns 5 and 6 show the percentage mean (MSE) and percentage standard deviation (SDSE) of the squared error of the Hopfield linear solver output relative to double-precision MATLAB quantity (‖Δ*H*‖). The results of Hopfield linear solver (matrix *H*) were compared against the results that were obtained using MATLAB's double precision pseudoinverse function. Per the error analysis in section 2.5.2 and the principles of stochastic computing (Alaghi and Hayes, 2013), progressive precision holds true for the proposed Hopfield solver. That is, as the number of clock ticks increases, the stochastic error asymptotically approaches zero. We can say from the results of Table 1 that the output matrix has a value which is quite close to its double-precision pseudoinverse counterpart in many cases.

Inputs that require low precision for computations (Experiments 1, 2, and 3 in Table 1) converge faster and show lower MSE in comparison to inputs that require higher precision (Experiments 9, 14, and 15 in Table 1). Note that the scenarios in which the Hopfield linear solver algorithm shows high MSE occur because the algorithm requires considerably more iterations to converge and precision greater than or equal to 10^−6^ to reach a solution. Since the proposed work is using stochastic computing, it would require at least 1 million ticks in the best-case scenario to represent a precision of 10^−6^ for a single value, as well as requiring more iterations to converge. While implementing the Hopfield solver on spiking neural substrate such as TrueNorth, the developer would have to consider this speed-accuracy tradeoff. For low-precision values, the Hopfield solver would converge faster, but many more ticks may be required for for high precision values.

### 3.2. Implementation analysis

When the firing rates of TrueNorth neurons saturate, the actual outputs of the Hopfield linear solver algorithm may no longer match the expected output; in fact, the difference may be quite large. However, for a large enough input scaling factor, the firing rates of neurons will be low enough so that they will never saturate.

**Figure 6:**
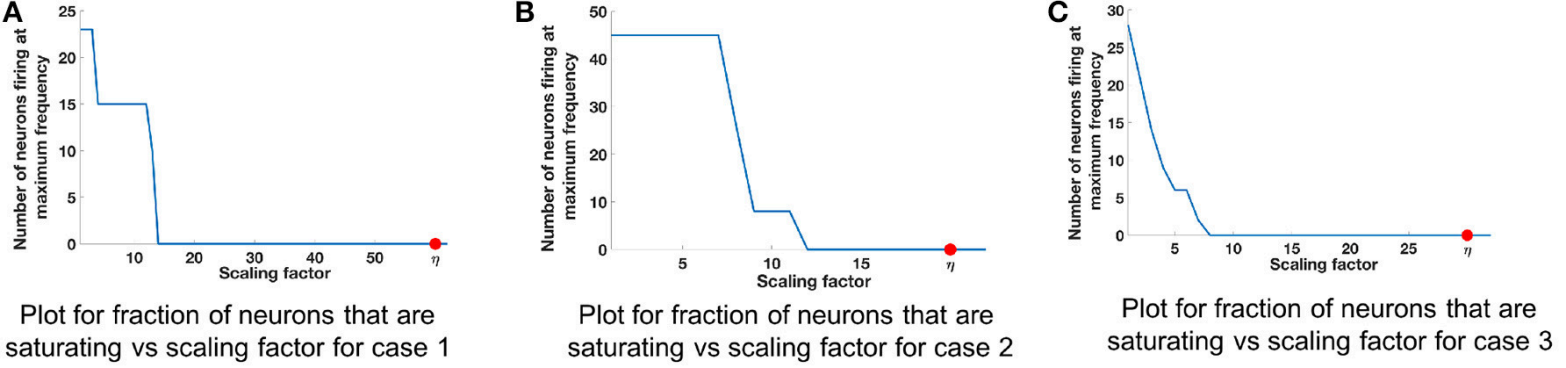
Comparison of scaling factor for different matrix structures. Table 2 lists the three different input matrices A and B. **(A)** Plot for fraction of neurons that are saturating vs. scaling factor for case 1. **(B)** Plot for fraction of neurons that are saturating vs. scaling factor for case 2. **(C)** Plot for fraction of neurons that are saturating vs. scaling factor for case 3.

We refer to cases 1, 2, and 3 in Table 2 for range analysis. Figures 6A–C show the plots for number of neurons that are saturating at maximum frequency vs. the scaling factor that was assigned to normalize the values of input. The neuron firing rates were collected using the corelet filter API which is a part of IBM TrueNorth's corelet programming environment (Amir et al., 2013). The firing rate of neurons was gathered for matrices A and B with different set of values, as shown in Table 2. The factor η (the scale factor bound calculated in section 2.2) proves that the computed values never saturate, irrespective of whether the computations are happening in the positive or negative domain. The bounds shown are high because the maximum value that every element in the matrix can have after the geometric series summation would be a multiple of $\sigma_{\min}^{-1}$. If the matrix *A* contains elements with very small magnitude then the term σ_min_ will be small as well; as a result we get a larger scale factor. The scenarios where η is close to the desired bound is when all of the elements in a matrix are the same and each element has values of high magnitude, similar to Case 2 in Table 2 (Figure 6B).

**Table 2:** Sample matrices for worked out examples.

**Case 1**	**Case 2**	**Case 3**	**Case 4**	**Case 5**
$A=\left[\begin{array}{ccc} 0.1 & 0 & 0\\ 0 & 0.1 & 0\\ 0 & 0 & 0.1 \end{array}\right]$	$A=\left[\begin{array}{ccc} 0.1 & 0.1 & 0.1\\ 0.1 & 0.1 & 0.1\\ 0.1 & 0.1 & 0.1 \end{array}\right]$	$A=\left[\begin{array}{ccc} 0.1 & -0.1 & 0.2\\ -0.2 & 0.1 & 0.1\\ 0.1 & 0.4 & -0.1 \end{array}\right]$	$A=\left[\begin{array}{cc} 0.08 & 8\\ -1 & 0.01 \end{array}\right]$	$A=\left[\begin{array}{cc} 0.8 & 1.25\\ 1 & 0.00008 \end{array}\right]$
$B=\left[\begin{array}{ccc} 1 & 0 & 0\\ 0 & 1 & 0\\ 0 & 0 & 1 \end{array}\right]$	$B=\left[\begin{array}{ccc} 1 & 1 & 1\\ 1 & 1 & 1\\ 1 & 1 & 1 \end{array}\right]$	$B=\left[\begin{array}{ccc} 1 & -1 & 1\\ -1 & 1 & 1\\ 1 & 1 & -1 \end{array}\right]$	$B=\left[\begin{array}{c} -4\\ 0.2 \end{array}\right]$	$B=\left[\begin{array}{c} 1\\ 1 \end{array}\right]$
η = 60	η = 20	η = 30	δ_hardcoded_ = 0.025% δ_spiking_ = 3.39%	δ_hardcoded_ = N/A δ_spiking_ = 0.8%

Cases 4 and 5 of Table 2 show the comparison of absolute errors when the same matrices are given as inputs, where *W*_ff_ and *W*_hop_ are either hard coded on TrueNorth or are supplied as spike train inputs. As per case 4, absolute error for hardcoded weights (δ_hardcoded_) is less than spiking weights (δ_spiking_) for same number of spike ticks. This is because hardcoding the weights gives us more control over precision when compared with spiking weights. In Case 5, δ_hardcoded_ cannot be computed because TrueNorth neuron's threshold parameter has a limited number of bits, so *W*_ff_ cannot be mapped onto the board using the technique of Algorithm 1. This problem does not occur with the spike train representation, as higher precision can be represented with longer duration.

### 3.3. Precision analysis

The goal of this section is to analyze the quantization error bound and stochastic error bound with the worst-case erroneous output of the Hopfield linear solver. We do so by injecting errors into the weight and input matrices and measuring the resulting error. We are evaluating whether the bounds are tight enough so that they are close to the worst-case erroneous Hopfield linear solver output. Initially, quantization error is introduced in the weight and input matrices and its effect is analyzed for the linear solver. Next, stochastic error is added to weight and input matrices, and the linear solver simulation results are compared with the stochastic bound that was derived in section 2.5.2. Despite being hard to predict, our simulations show that the stochastic error can reach an average of 70% of the bound demonstrating sufficient agreement between the analysis and simulated data.

In order to evaluate the effect of quantization error, the output of the Hopfield network is compared for two cases. In the first case, we provide the exact input and weights to the Hopfield network and calculate the output. In the second case, we introduce some error to the input and the weights equal to the maximum quantization error, and again evaluate the output of the network. Finally, we compare these two outputs and calculate the output error.

The error evaluation results are shown in Table 3 for the cases presented in Table 2. These results show that for all cases, the error remains below the estimated bound. In addition, the error in Case 1 is close to the estimated (91%) bound, which indicates that the bound is tight enough to be useful. It is also important to note that in Case 2, where the matrix *A* has singular values equal to 0, the error remains below the bound. Thus, even though there are scenarios where our analytical approach does not provide a guarantee, in practice, the estimated error bound holds.

**Table 3:** Quantization error simulation results.

	**Error relative to the bound (%)**
Case 1	90.75
Case 2	5.13
Case 3	38.63
Case 4	13.89
Case 5	24.86

Later, we evaluate the stochastic error bound using a similar method as was used for quantization error. However, due to the randomness of the stochastic error, we repeat each test 100 times and report both the average over all repetitions as well as their maximum. As mentioned in section 2.5.2, the calculated bound does not define an absolute upper limit for each repetition, but rather a bound for their average. In other words, we expect the average error over multiple runs to be smaller than the estimated bound, but the maximum value of the error could exceed the bound.

**Figure 7:**
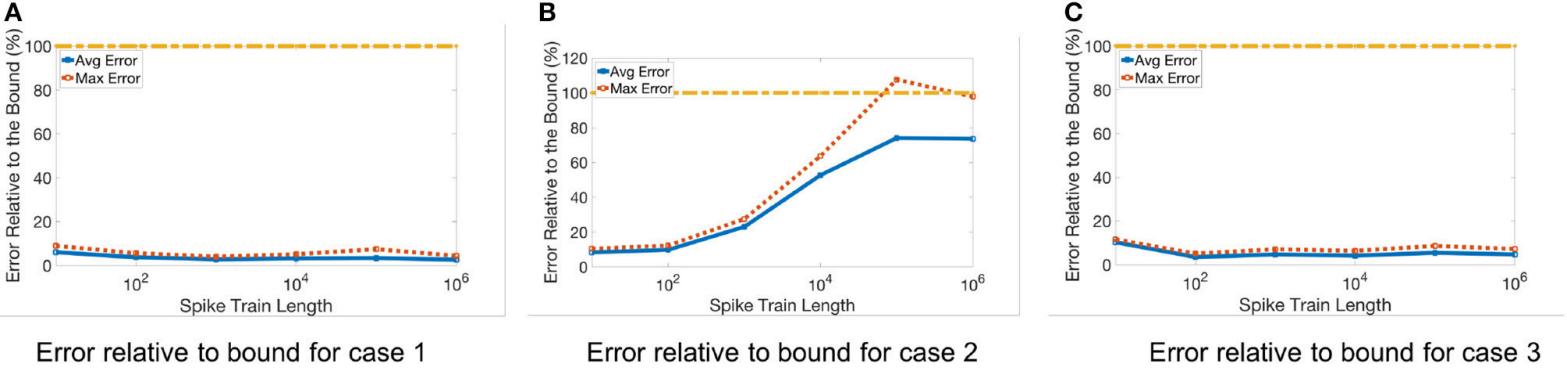
Stochastic error for different spike train lengths. Table 2 lists the three different input matrices A and B. **(A)** Error relative to bound for case 1. **(B)** Error relative to bound for case 2. **(C)** Error relative to bound for case 3.

The results for three representative cases (Cases 1, 2, and 3, from Table 2) are shown in Figure 7 for different spike train lengths. The average error always remains below the bound, but the maximum error for Case 2 exceeds the bound at some points. Furthermore, the gap between the bound and the average error is larger than when we only consider the quantization error. This is due to the unpredictable nature of the stochastic error resulting in a looser stochastic error bound.

Simulations of the quantization and stochastic errors show that the proposed bounds can provide reasonable upper limits for the error of the Hopfield network implemented on a neural network hardware. Therefore, these bounds can be used to gain insight into the precision results of this network for any set of input and weights, before running the algorithm. In addition, they can be used to allocate appropriate resources in order to achieve a specific output precision.

### 3.4. Architecture-application analysis

Prior work Shukla et al. (2017) showed how these linear solvers can be used to compute transformation matrices for applications such as inverse kinematics, object tracking and optical flow. To analyze the proposed Hopfield linear solver in a practical implementation scenario, we tested the proposed work for Lucas-Kanade based optical flow application that has a setup similar to the one shown in Figure 2B and described in the prior works (Esser et al., 2013; Shukla et al., 2017). The image in Figure 2B is a grayscale image in which high intensity pixels are represented with a value of 1 and low-intensity pixels are represented with 0. The two black bars in the figure have a pixel width of 5 pixels. the resolution of the image was set at 240-by-360 pixels which is same as QVGA format videos. The horizontal and vertical bars were initially positioned at the center along height and width of the image, respectively. The two lines intersected at the center of the image. The sequences of images are streaming in to the hardware at 30 frames per second. For the first set of frames, the horizontal bar is moving upwards, and the vertical bar is moving toward left. In the implementation, the frame size of QVGA video was first reduced by a factor of 4 to 120-by-180 pixels, then a 5-by-5 pixels convolutional operation was applied to it.

The implementation of a Hopfield linear solver in such a setup is challenging since the Hopfield neural network (*W*_ff_ and *W*_hop_) weights change continuously. Also, in this setup there is no training or testing data involved. The goal here is to compute the results online by just looking at the streaming input values without any prior knowledge of the experiment or scenario. We observe additional benefits by deploying multiple linear solvers in parallel since we have to calculate pseudoinverse for multiple different locations on the image at the same time. These experiments give us better insights with respect to selecting TrueNorth as a potential substrate for deployment of such algorithms, and provides a vehicle for energy analysis when compared with more traditional approaches. In this experiment we measure the motion vector error against the baseline, but have also utilized an approximately correct metric: as long as the solver correctly detects flow in one of eight possible ordinal and cardinal directions, we count it as correct. The velocity of the movement of two bars is calculated by solving for *X* in the equation *AX* = *B*. Matrix A contains partial derivatives of initial image frame with respect to directions x and y around pixel qi. This is represented by terms *I*_*x*_(*qi*) and *I*_*y*_(*qi*), in Equation (44). Matrix B contains partial derivatives of pixel positions between initial image frame and image frame at time t around pixel qi. This is represented by terms *I*_*t*_(*qi*), in Equation (45). After implementing matrix division, output matrix *X* will report the speed and direction of the image pixels, by computing the pseudoinverse of matrix *A*.

(44) $$\begin{array}{l} A=\left[\begin{array}{cc} I_{x}\left(q1\right) & I_{y}\left(q1\right)\\ I_{x}\left(q2\right) & I_{y}\left(q2\right)\\ \vdots & \vdots \\ I_{x}\left(qn\right) & I_{y}\left(qn\right) \end{array}\right] \end{array}$$

(45) $$\begin{array}{l} B=\left[\begin{array}{c} -I_{t}\left(q1\right)\\ -I_{t}\left(q2\right)\\ \vdots \\ -I_{t}\left(qn\right) \end{array}\right] \end{array}$$

In the proposed setup, we can have multiple input matrices A and B (see Equation 3), that are independent of each other, since the convolution operation can operate on separate and independent patches of image at the same time. The results of these independent convolutions can be streamed as different input matrices A and B. As a result, we can have multiple independent linear solvers running in parallel to compute different pseudo-inverses for these different input matrices. For a frame of size 120-by-180 pixels, linear solver implementation processed 9,800 pixels of a single frame to predict the motion vectors.

Using the optical flow implementation described above, we compare the power and energy consumption of TrueNorth based linear solver implementation with more traditional approaches like QR inverse algorithm on Virtex-7 FPGA (xc7vx980t) and on an ARM cortex A15 mobile processor.

On TrueNorth we can implement 392 instances of the Hopfield linear solver that operate in parallel independent from one another. These 392 instances required 4,092 cores of the available 4,096 cores and can process roughly 9,800 pixels for predicting the motion vectors. Therefore, we would need to compute optical flow motion vectors in the specified scenario in batches of two streaming input pixels for a single 120-by-180 pixels frame.

To maintain the throughput of 30 FPS for 9,800 pixels we needed an 8-core ARM chip operating at 2.5 GHz. For the same FPS and pixel count instantiate 32 instances of QR inverse algorithm on Virtex-7. A detailed discussion about each of the implementation technique is presented as follows:

***TrueNorth:*** We have implemented 392 instances of the Hopfield linear solver which operate in parallel, independent from one another. These 392 instances required 4,092 cores of the available 4,096 cores. The power consumption values were reported from IBM's test and development board. For a supply voltage of 0.8 V and 1KHz operating frequency, the scaled leakage power of our implementation is 46.31 mW and the scaled active power 18.67 mW. Since the goal is to implement optical flow at 30 FPS, we increase the operating frequency of TrueNorth NS1e hardware to 9KHz and report a linearly scaled active power of 168.03 mW for these experiments.
***Virtex-7 FPGA:*** The QR inverse algorithm was implemented using the matrix algebra libraries present in Xilinx Vivado HLS (Xilinx, 2014) and the frequency of the platform was set at 20 MHz. Power analysis of the following implementations were done using Xilinx Power Estimator tool (Xilinx, 2017). For 32 parallel instances of QR inverse solver the total power consumption is 1.881W with a static power consumption of 383 mW.
***8-core ARM A15 processor:*** The QR inverse algorithm was implemented using the C++-based eigen library (Guennebaud et al., 2010). Simulations for matrix inversion were done using gem5 (Binkert et al., 2011) in the system emulation mode and the power consumption details were collected using *McPat* (Li et al., 2009). We used the ARM A15 configurations for Gem5 and McPat simulations that have been presented in (Endo et al., 2015). For processing the QR inverse algorithm, an octa-core ARM Cortex A-15 chip consumed 2.55 W of power which includes 93.5 mW of static power.

**Figure 8:**
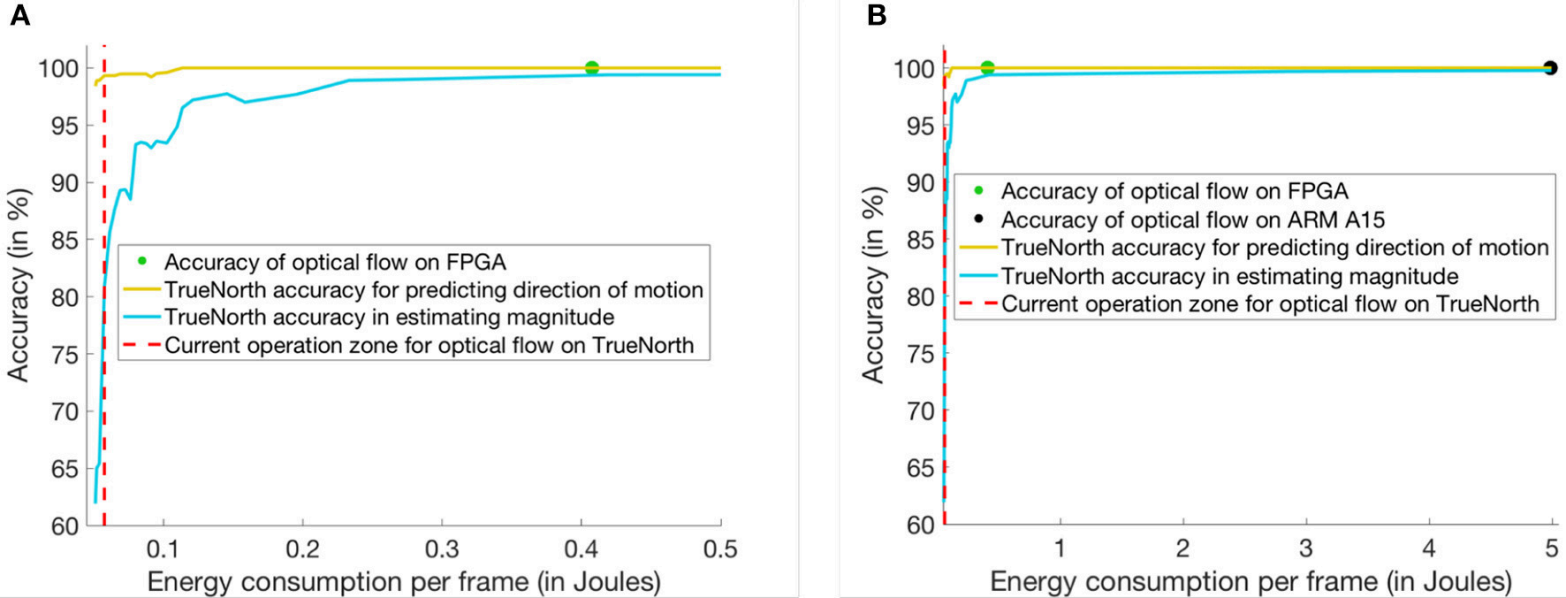
This figure shows a comparison between three different implementation techniques for matrix inversion. Y-axis of the plot shows the percentage accuracy in predicting the motion of bars for optical flow. And, X-axis of the plot shows the energy consumed per frame (in Joules) for optical flow. **(A)** Comparison of power consumed between FPGA and TrueNorth hardware. **(B)** Comparison of power consumed between ARM, FPGA and TrueNorth hardware.

Figure 8 shows the comparison of energy consumption and time elapsed for computation on three different hardware platforms. Since TrueNorth can perform computations on 9,800 pixels at a time, it would have to time-multiplex 120-by-180 pixels into two batches to perform the linear solver operation. At 9 KHz frequency each time multiplexed batch would need a maximum of 150 time ticks for computing the inverse on a portion of the image. After 150 ticks, the accuracy of predicting the direction in optical flow is 99.33% and the speed of motion can be estimated with an accuracy of 80.9%. As per the plots in Figure 8A,B, the TrueNorth-based linear solver is more energy efficient than the ARM or FPGA implementations. For both the FPGA- and ARM-based QR inverse solvers, the accuracy is 100% as they are using floating point units for computation. The TrueNorth-based linear solver consumes 0.0575 J of energy per frame, the FPGA consumes 0.4074 J of energy per frame and, the ARM processor consumes 4.986 J of energy per frame. On the other hand, the accuracy of TrueNorth depends on how many ticks it requires. As a result, if TrueNorth is operated for more ticks, the solution achieves higher accuracy but consumes more energy.

## 4. Discussion

To the best of our knowledge, this paper is the first attempt to formalize a mathematical framework for determine scaling factors and error bounds when deploying a recurrent numerical solver on limited-precision neural hardware. The proposed research developed a mathematical and algorithmic framework for calculating generalized matrix inverses on this hardware platform. Apart from using the proposed algorithm for real-time robotics applications, it could also be used for on-chip training of multi-layered perceptrons and extreme-learning machines (Tang et al., 2016) for a variety of classification and regression based tasks. We validate the mathematical model using a Hopfield network-based linear solver that has been implemented on the IBM TrueNorth spiking neural substrate. Our empirical results show that the analytic bounds are never violated for the scenarios evaluated.

### 4.1. Summary of experiments and results

First, section 3.1 compares the results of proposed linear solver against MATLAB's double precision pseudo-inverse function. Results presented in Table 1 suggests that a stochastic-computing implementation can produce an output matrix that are quite close to their double-precision pseudoinverse counterparts in many cases. However, the developer would have to keep in mind speed-accuracy tradeoff. For low precision values, the Hopfield solver would converge faster, while many more ticks would be required for high precision values.

Second, section 3.2 presents the range analysis of Hopfield linear solver. We can guarantee that the proposed scaling factor will keep the firing rates of neurons low enough that they never saturate. Similarly, in section 3.3, we validate the bounds that were proposed in precision analysis. For quantization error, the experimental errors can get very close to estimated (91%) bound, indicating that the bound is tight enough to be useful. For stochastic errors, the average of experimental error always remains below the bound.

Finally, section 3.4 compares the TrueNorth-based Hopfield linear solver against standard QR inverse algorithms that were implemented on the ARM processor and in FPGA. Experiments with the optical-flow application showed the energy benefits of deploying a reduced-precision and energy-efficient generalized matrix inverse engine on the IBM TrueNorth platform. Since TrueNorth architecture was designed to be low power, deployment of multiple linear solvers running in parallel could give a 10 × to 100 × improvement in terms of energy consumed per frame over FPGA and ARM core baselines.

### 4.2. Extending hopfield neural network based linear solver to other hardware substrates

Sections 2.3.2 and 2.4 present algorithms that can compute matrix inverses using concepts from stochastic computing (Gaines, 1967). The proposed algorithms can be extended to other spiking and non-spiking hardware substrates that have the ability to perform stochastic computing and provides the capability to have recurrent neural network connections. Prior work such as Smithson et al. (2016), Thakur et al. (2016), and Cassidy et al. (2013) show that digital spiking neural substrates can perform stochastic computing. We can also perform stochastic computing on non-spiking hardware substrates such as FPGAs (Li et al., 2016), FinFETs (Zhang et al., 2017), and magnetic-tunnel-junction (Lv and Wang, 2017). These technologies provide us with a promising opportunity to implement linear solvers based on Hopfield neural networks while being energy-efficient and operate at a higher frequency. Developers would have to keep in mind that the proposed linear solver is performing lossless addition (Figure 5C). When a neuron receives spikes from multiple inputs at the same, its membrane potential increases by the same amount as the number of input spikes it has received at that time tick. The membrane potential decreases by one after the neuron fires. Scaling factor η that was derived in Equation (19) and Claim 1 guarantees that even with a lossless addition present in the equations, the intermediate computation will never saturate.

### 4.3. Future work

In future work, we will look into speeding up the computation by using a population coding scheme for encoding values to spikes. Our current implementation uses a rate coding technique for encoding values with a single neuron. Considering the resources that we have available on TrueNorth board, a population coding scheme could perform computations in parallel, hence reducing the time to solution.

Large scaling factor values may end up resulting in longer computation time, since we end up requiring more ticks to represent the scaled values accurately. Alternatively, tight scaling factors that still avoid saturation require computation of the matrix pseudoinverse, using external hardware. This complicates deployment of the Hopfield solver in scenarios where the matrix changes over time. Developing tighter bounds, especially ones that are easier to compute online, would avoid these problems.

Finally, in the proposed architecture in this paper the term α was precomputed. As part of our future work, we would like to create a TrueNorth based framework where α could be computed dynamically via spikes.

## Author contributions

RS was responsible for preparing the manuscript, scaling analysis of linear solver, coming up with the algorithm for hardware implementation of linear solver and all of the experiments related to hardware (TrueNorth, FPGA and ARM) analysis and hardware implementation. RS also did the scaling factor based experiments. SK was responsible for mathematical theory of error analysis and precision based error analysis. EJ did the implementation of algorithm on TrueNorth hardware. JL, and ML were the ones that guided the project, came up with initial ideas. SW suggested corrections for mathematical theorem and proofs, as well as did rewritings in the manuscript.

### Conflict of interest statement

ML has financial interest in Thalchemy corp. and is co-founder of the said corporation. Thalchemy corp. was not at all involved in this research project in any form. The other authors declare that the research was conducted in the absence of any commercial or financial relationships that could be construed as a potential conflict of interest.
